# Effect of diode laser and hyaluronic acid on occlusion of dentinal tubules: an in-vitro study on human dentin

**DOI:** 10.1186/s12903-026-09733-1

**Published:** 2026-09-09

**Authors:** Riham Hisham Mahmoud, Basma Hosny Mohamed, Rehab Khalil Safy, Mohamed Mostafa Abdel Moaty

**Affiliations:** 1 Suez Canal University, Ismailia, Egypt; 2 Sinai University, Ismailia, Arab Republic of Egypt

**Keywords:** Dentin hypersensitivity, Dentinal tubule occlusion, Diode laser, Hyaluronic acid, Sodium fluoride varnish, Desensitizing agents, pH cycling, Human dentin, In vitro study

## Abstract

**Background:**

Dentin hypersensitivity (DH) is a common clinical condition characterized by short, sharp pain arising from exposed dentinal tubules. Occlusion of dentinal tubules remains a widely accepted approach for managing DH in dental practice. This in vitro study aimed to evaluate and compare the effects of sodium fluoride, hyaluronic acid, diode laser, and their combinations on dentinal tubule occlusion in human dentin.

**Methods:**

Sixty extracted human impacted third molars were sectioned into dentin discs and randomly allocated into five groups (*n* = 12): sodium fluoride varnish (M1), hyaluronic acid (M2), diode laser irradiation (M3), hyaluronic acid + diode laser (M4), and hyaluronic acid + sodium fluoride varnish (M5). All specimens were treated after initial smear layer removal with 17% EDTA. The surface area percentage of open dentinal tubules was evaluated using Environmental Scanning Electron Microscopy (ESEM) and ImageJ software at three time points: baseline (T0), after treatment (T1), and after pH cycling (T2). Data were analyzed using a two-way repeated-measures ANOVA to assess the effects of treatment modality, time, and their interaction, followed by Bonferroni post hoc tests (α = 0.05).

**Results:**

Repeated-measures ANOVA revealed a statistically significant effect of treatment modality (*p* < 0.001), time (*p* < 0.001), and a significant treatment × time interaction (*p* < 0.001), indicating that changes over time differed among groups. At baseline (T0), no significant differences were observed among groups (*p* = 0.190). Following treatment (T1), the lowest mean surface area of open dentinal tubules was recorded in the hyaluronic acid + diode laser group (M4), while the highest was observed in the sodium fluoride group (M1). After pH cycling (T2), all groups showed an increase in tubule openness; however, M4 maintained the lowest values, indicating superior resistance to acidic challenge. Pairwise comparisons demonstrated statistically significant differences among most groups, except between M2 and M5.

**Conclusions:**

Within the limitations of this in vitro study, all tested modalities were effective in reducing the surface area of open dentinal tubules to varying degrees. The combination of hyaluronic acid and diode laser demonstrated the most pronounced occluding effect, indicating enhanced efficacy compared with the individual treatments. However, given the surface-based and laboratory nature of the assessment, further in vivo studies are required to confirm the clinical relevance and long-term stability of these findings.

**Supplementary Information:**

The online version contains supplementary material available at https://doi.org/10.1186/s12903-026-09733-1.

## Introduction

Dentin hypersensitivity (DH) is a prevalent condition characterized by a short, sharp pain arising from exposed dentin in response to external stimuli [1]. Dentin is highly sensitive due to its close structural and functional relationship with the dental pulp; therefore, its exposure allows external stimuli to trigger pulpal nerve responses [2]. Several mechanisms have been proposed to explain the pathophysiology of DH, including the neural theory and the odontoblastic transduction theory. However, the hydrodynamic theory remains the most widely accepted explanation, suggesting that fluid movement within dentinal tubules stimulates mechanoreceptors at the pulpal nerve endings [3]. Currently, various materials and techniques are utilized for such purpose such as: potassium-based agents, oxalates, resin-based sealers, bioactive materials. Fluoride derivatives work by depositing a calcium fluoride layer that acts as a barrier, this protective layer seals the opening of dentinal tubules and decreases hypersensitivity. However, fluoride only offers a transient pain relief since it is affected by the acidic oral environment and is quickly eliminated by brushing or wiping the tooth surface. As a result, its longevity is uncertain [4]. Also, it has been accepted that the widespread use of fluoride toothpastes is related to the current levels of fluorosis observed in the populations exposed to it. Based on such drawbacks, searching for other alternatives was necessary. Hyaluronic acid (HA) is a naturally occurring, non-sulfated glycosaminoglycan widely present in biological tissues. It exists in a broad range of molecular weights, from low to high, with each fraction demonstrating distinct biological activities [5]. In the present study, high–molecular-weight HA was specifically utilized. It is a critical element of the cementum, alveolar bone, gingiva, and periodontal tissues. It has several physiological and structural functions within these tissues and plays an important role in their development and regeneration [6]. The viscoelastic characteristics of HA are also significant in preventing the penetration of bacteria and viruses into both mineralized and non-mineralized tissues [7]. Therefore, its utilization as a biomaterial in treatment of periodontal pockets due to its osteogenic, anti-inflammatory and antibacterial effects was recommended [8]. Based on such fact, it may offer potential benefits in dentin hypersensitivity management [9]. On the other side, Lasers have been introduced as another option for in-office treatment of DH, where a continuous wave is produced by low-power output lasers like diode lasers without heating the substrate [10]. Currently, diode lasers are hypothesized to treat DH by reducing nerve transmission and dissolving the dentin structure, which blocks the dentinal tubules [11]. In addition, different types of lasers have been used in the management of dentin hypersensitivity, including low-power lasers that act through photobiomodulation and high-power lasers such as Nd: YAG and Er: YAG, which can induce surface melting and recrystallization of dentin, resulting in dentinal tubule occlusion. Laser therapy may also provide immediate pain relief. Furthermore, laser-induced surface modifications may improve the retention of desensitizing agents when used in combination; however, evidence supporting a definitive synergistic effect remains limited. Therefore, this study was concerned with evaluating the effect of diode laser hyaluronic acid and their combination in comparison to the conventionally used sodium fluoride on occlusion of dentinal tubules. The research hypothesis was that there would be a significant difference between sodium fluoride, hyaluronic acid, diode laser, and their combinations in their ability to occlude dentinal tubules, with the combined application of hyaluronic acid and diode laser expected to demonstrate a superior occluding effect due to a potential synergistic interaction. The null hypothesis was that there would be no significant difference among the tested groups.

## Materials and methods

### Materials

Materials’ description, composition, manufacturers and batch numbers are presented in Table 1. 

**Table 1:** The materials’ brand name, description, composition, manufacturers and batch numbers

Materials	Description	Composition	Manufacturers	Batch numbers
EDTA sol.	Chelating agent	17% Ethylene diamine tetra acetic acid, Ammonia, water	Metabiomed, Chungbuk, Republic of Korea	MDL201
Profluorid varnish	Fluoride varnish	5% Sodium fluoride, Xylitol	Voco, Cuxhaven, Germany	2,038,399
Gengigel	Hyaluronic acid hydrogel	0.8% Sodium Hyaluronate. Other ingredients: Aqua, Xylitol.	Ricerfarma, Milano, Italy	SF3001
Demineralizing solution	Demineralizing medium	2.2 mM CaCl2, 2.2 Mm NaH2PO4, 50 mM acetic acid.	Al-Azhar University, Egypt.	---
Remineralizing solution	Remineralizing medium	1.5 mM CaCl2, 0.9 mM NaH2PO4, 0.15 M KCl.	Al-Azhar University, Egypt.	---

### Methods

#### Study design

The present study was an in vitro experimental study with five groups. Ethical approval was waived by the Ethical Committee of the Faculty of Dentistry, Suez Canal University, Egypt (No. 556/2022), as the study was conducted on extracted human teeth with no patient identifiers.

#### Sample size calculation

This study aimed to evaluate the effects of sodium fluoride, diode laser, hyaluronic acid, and their combinations on dentinal tubule occlusion. The sample size was calculated using G*Power software (version 3.1.9.7) [12].Based on a two-way repeated-measures ANOVA, an effect size (f) of 0.27 estimated from a previous literature [13] an alpha (α) level of 0.05, and a power (1-β) of 0.90, the minimum total sample size required was determined to be sixty specimens (n = 12 samples per group). The primary outcome variable for this calculation was the surface area percentage of open dentinal tubules (DT %) at the final time point (T2).”

#### Teeth selection

Sixty impacted mandibular third molar teeth extracted from patients in the age range between 20 and 40 years old were collected. All teeth were free of caries, cracks, abrasions, stains and demineralization. Teeth were thoroughly washed with water, scaled with periodontal scaler then examined for freedom of cracks or defects using a magnifying lens x3. Then, all the collected teeth were stored in distilled water with 0.2% thymol for no longer than one month to inhibit microbial growth [14].

#### Samples preparation

All teeth were randomly allocated into five main groups (*n* = 12) according to the application of different treatment modalities. The occlusal enamel of all teeth was removed using an automated diamond saw (Isomet^®^ 4000 Linear Precision Saw: Buehler Ltd., Lake Bluff, USA) under copious water cooling, in a direction perpendicular to the long axis of the teeth. Preparation was done by removal of the enamel till 1.5 mm beneath the central groove, using size 2 round carbide bur, an indentation of 1.5 mm depth in dentin was prepared. The depth of the indentation was guided using a rubber stopper mounted to the shaft of the round bur. Preparation was done utilizing the same diamond disc till indentation disappeared [15]. Then, a second cut was made perpendicular to the long axis of each tooth to obtain dentin discs of 2 mm thickness. The dentin surfaces of all samples were standardized by polishing with 1200-grit silicon carbide paper for 60 s under running water to obtain a smooth, flat, and uniform surface. Afterward, dentin discs were immersed in 17% EDTA solution for 2 min [16] to open the dentinal tubules to ensure that the prepared dentin surface was free of any smear layer or smear plugs [17] then they were thoroughly rinsed with de-ionized water.

### Environmental scanning electron microscope evaluation

All images were acquired at a fixed magnification of ×2000 to ensure consistency and optimal visualization of dentinal tubules [18]. Three representative fields were captured per specimen at each time point (T0, T1, T2) from standardized random areas to account for dentin surface heterogeneity [19, 20]. For repeated measurements, efforts were made to acquire images from comparable regions of interest across time points; however, to minimize sampling bias, standardized random fields within predefined areas were selected. The mean value of the three images was used for statistical analysis.

Environmental scanning electron microscopy (ESEM) imaging was performed without conductive coating to enable repeated evaluation of the same specimens. Imaging was conducted using an environmental scanning electron microscope (FEI Quanta FEG 250, Thermo Fisher Scientific, Hillsboro, OR, USA) under standardized operating conditions, including an accelerating voltage of 20 kV, a working distance of 15.6 mm, and backscattered electron detection (BSED) mode. Imaging was performed in low-vacuum (environmental) mode, with chamber pressure maintained according to manufacturer-recommended conditions suitable for hydrated biological samples, ensuring preservation of dentin surface characteristics during repeated imaging.

ESEM evaluation and image analysis were performed by a single trained examiner using a standardized protocol. The examiner was blinded to group allocation and time points during analysis to minimize bias, and all measurements were conducted using a strictly predefined and uniform workflow.

Image analysis was performed using ImageJ software (ImageJ 1.60, Scion, Frederick, MD, USA). All images were first calibrated using the embedded scale bar. A standardized and uniform thresholding protocol with fixed parameters was applied consistently across all images to differentiate open dentinal tubules from intertubular dentin based on grayscale intensity. Binary conversion of the images was then performed, followed by quantification of the surface area of open dentinal tubules using the “Analyze Particles” function. Results were expressed as a percentage (%) of the total analyzed surface area. The same analysis workflow and identical parameters were applied to all specimens to ensure methodological consistency, reproducibility, and to minimize operator-related variability and potential analytical bias. Representative images illustrating the thresholding and analysis procedure are provided in Supplementary Figure S.

### Samples treatment

Treatment of the samples according to the occluding protocol was carried out as follows: in M1 group (sodium fluoride group, used as an active comparator), dentin discs were treated with 5% NaF varnish (Profluorid varnish, Voco, Cuxhaven, Germany). A thin and uniform layer of NaF varnish was applied on the dentin disc top surface, left for 20 s, and then air-dried for one minute. In M2 group, dentin discs were treated with hyaluronic acid gel (Gengigel^®^, Ricerfarma, Milano, Italy), where a thin uniform layer was applied for 2 min with gentle rubbing and then left undisturbed to dry. In M3 group, dentin discs were treated with a diode laser (Epic X, Biolase, California, USA) using a wavelength of 980 nm and a 320 μm fiber optic tip, operating in continuous mode at an output power of 1 W. Laser irradiation was applied for 30 s at a fixed distance of 1 mm from the dentin surface under standardized conditions. The estimated spot size at the dentin surface was approximately 0.32 mm in diameter, corresponding to an irradiation area of approximately 0.08 cm². Accordingly, the total energy delivered was 30 J, and the calculated energy density was approximately 375 J/cm² [21]. In M4 group, hyaluronic acid gel was applied followed by diode laser irradiation as previously described. Similarly, in M5 group, hyaluronic acid gel was applied followed by 5% NaF varnish as described above. All treatments were applied once at baseline with no re-application or additional waiting intervals. Subsequently, the surface area of open dentinal tubules was evaluated using ESEM and ImageJ analysis at T1.

### pH cycling

A 20 mL of a demineralizing solution (2.2 mM CaCl2, 2.2 Mm NaH2PO4, and 50 mM acetic acid adjusted to a pH of 4.8) for 8 h was utilized. Also, 20 mL of a remineralizing solution (1.5 mM CaCl2, 0.9 mM NaH2PO4 and 0.15 M KCl adjusted to a pH 7.0) for 16 h was utilized [22]. The solutions were daily renewed and the samples were rinsed with deionized water after each immersion. The pH values of the demineralizing solutions were monitored daily by pH meter. The.

pH cycling protocol was carried out for 14 days at room temperature. All solutions were freshly prepared before each phase of the pH cycle and renewed daily to inhibit saturation [23]. Afterwards, surface area of open DT was reassessed using ESEM at T2 and image analysis was carried out using Image J, as mentioned before. Comparison of the surface area of open DT percentage was made for each sample after the three times of examination by using computer-assisted digital image analysis (*Image J 1.60*,* Scion*,* and Frederick*,* MD*,* USA*) [16].

### Statistical analysis

All data were calculated, tabulated, and statistically analyzed using suitable statistical tests as follows; A normality test (Shapiro-Wilk) was done to check the normal distribution of the samples. Descriptive statistics were calculated in the form of Mean ± Standard deviation (SD). Two-way repeated-measures ANOVA was used to compare between the groups, time and their interactions under study. Bonferroni post hoc test was performed for pairwise comparisons among the groups. P value ≤ 0.05 is considered statistically significant. Statistical analysis was performed using the computer program SPSS software for Windows version 26.0 (Statistical Package for Social Science, Armonk, NY: IBM Corp) at significant levels < 0.05 (P- Value).

## Results

### Environmental scanning electron microscope results

The data presented in Table 2 illustrate the effect of different treatment modalities on the mean surface area percentage of open dentinal tubules (DT) across the three time points of evaluation (T0, T1, and T2). Normality of data distribution was assessed using the Shapiro–Wilk test, which confirmed that the data were normally distributed (*p* > 0.05). Two-way repeated-measures ANOVA revealed a statistically significant main effect of treatment modality (*p* < 0.001), a significant effect of time (*p* < 0.001), and a statistically significant treatment × time interaction (*p* < 0.001). This significant interaction indicates that the pattern of change in dentinal tubule openness over time was dependent on the type of treatment applied.

**Table 2:** Mean ± standard deviation (SD) of the surface area percentage of open dentinal tubules (%) for different treatment modalities at baseline (T0), after treatment (T1), and after pH cycling (T2)

Groups	Baseline (T0)	After different treatment modalities (T1)	After pH cycling (T2)	Mean
	Mean ± SD	Mean ± SD	Mean ± SD	
Sodium fluoride (M1)	14.86 ± 3.06^ab^	11.22 ± 0.97^c^	13.05 ± 1.55^b^	13.04^A^
Hyaluronic acid (M2)	13.60 ± 2.92 ^b^	6.79 ± 1.83 ^e^	8.68 ± 1.63^d^	9.69^C^
Diode laser (M3)	14.97 ± 2.60^ab^	8.84 ± 2.21^d^	10.06 ± 1.08^c^	11.29^B^
Hyaluronic acid + Diode laser (M4)	16.32 ± 2.65^a^	4.78 ± 1.33^f^	6.45 ± 1.15^e^	9.18^C^
Hyaluronic acid + Sodium fluoride (M5)	15.01 ± 1.88^ab^	6.71 ± 1.63^e^	8.08 ± 1.71^d^	9.93^C^
Mean	14.95^A^	7.67^C^	9.26^B^	
Two-way repeated-measures ANOVA				
Groups	< 0.001**			
Time	< 0.001**			
Group × time	< 0.001**			

Different lowercase letters indicate significant differences between groups at the same time point, while different uppercase letters indicate significant differences between time points within the same group (*p* < 0.05)

**; mean significant difference at *P* < 0.05

**Fig. 1:**
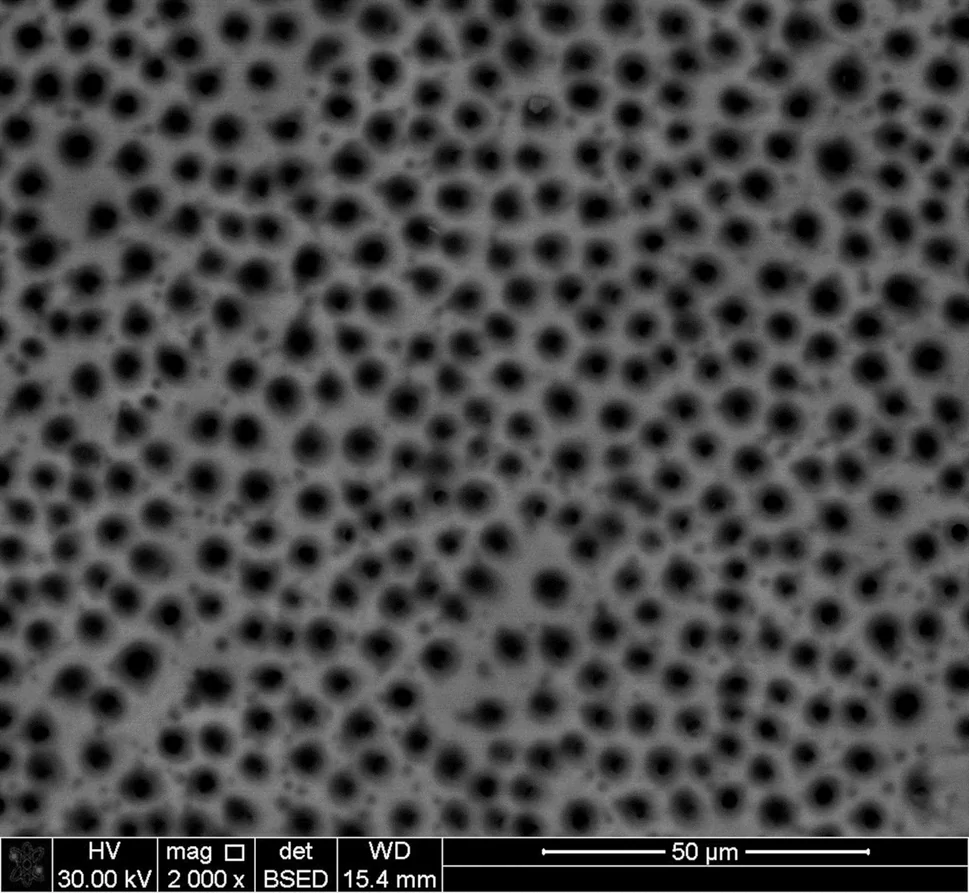
Representative ESEM photomicrograph (×2000) for dentin surface at baseline (T0), Scale bar = 50 μm

At baseline (T0), there was no statistically significant difference among all tested groups (*p* = 0.190), confirming the effectiveness of the standardization protocol. ESEM photomicrographs of all groups at this stage revealed widely open, patent dentinal tubules following EDTA treatment, presenting a characteristic spongy appearance with round and oval tubule morphology (Fig. 1).

Following the application of different treatment modalities (T1), a reduction in the surface area of open dentinal tubules was observed in all groups. The highest mean value (indicating the least occlusion) was recorded in the sodium fluoride group (M1) (11.22 ± 0.97), followed by the diode laser group (M3) (8.84 ± 2.21), the hyaluronic acid group (M2) (6.79 ± 1.83), and the hyaluronic acid + sodium fluoride group (M5) (6.71 ± 1.63). The lowest mean value (indicating the greatest degree of occlusion) was observed in the hyaluronic acid + diode laser group (M4) (4.78 ± 1.33). Post hoc analysis demonstrated statistically significant differences between M1, M3, and M4, while no significant difference was found between M2 and M5 (*p* < 0.001).

**Fig. 2:**
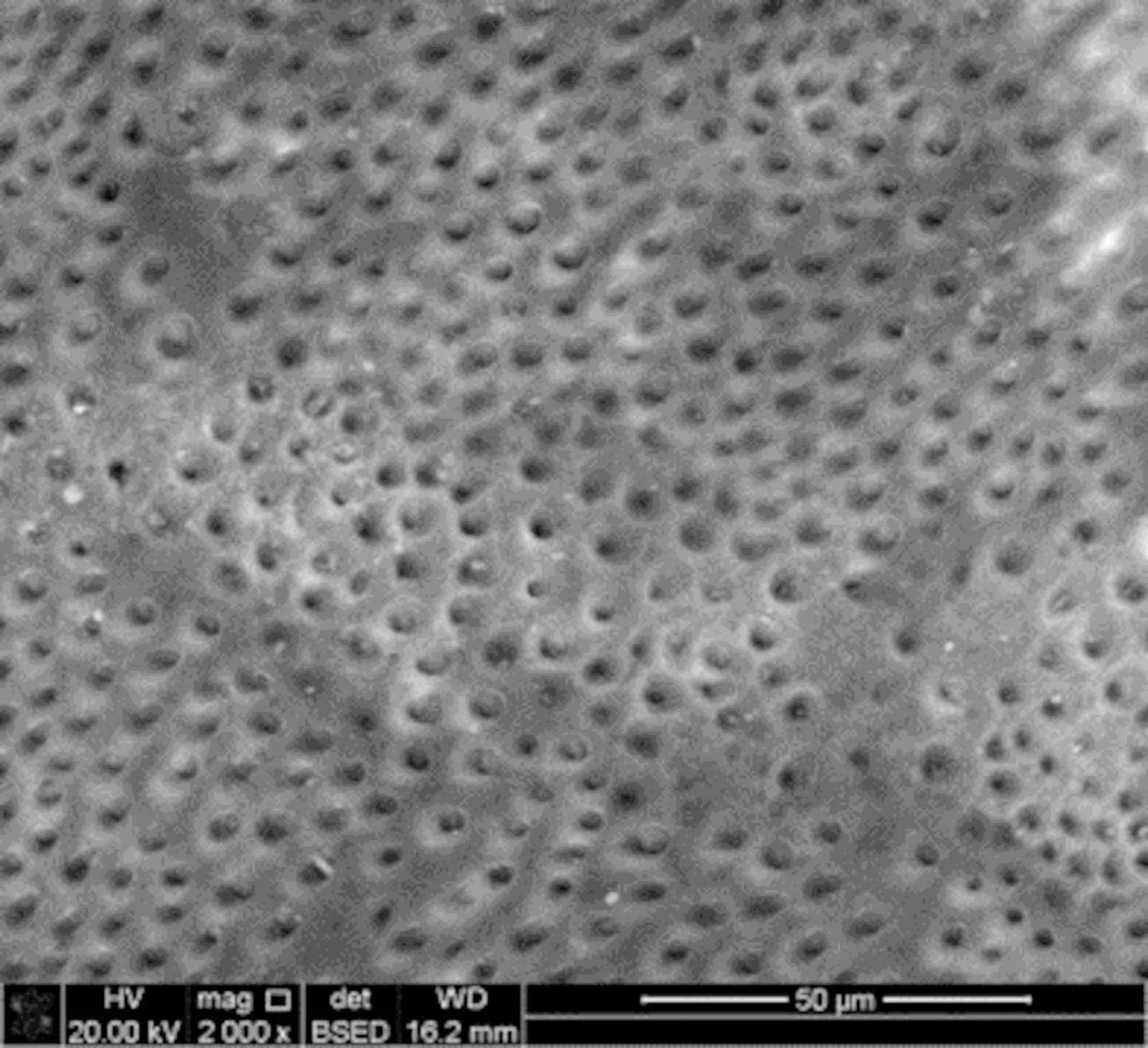
Representative ESEM photomicrograph (×2000) for dentin surface after sodium fluoride treatment (M1) at T1 (after treatment), showing partially occluded dentinal tubules, Scale bar = 50 μm

**Fig. 3:**
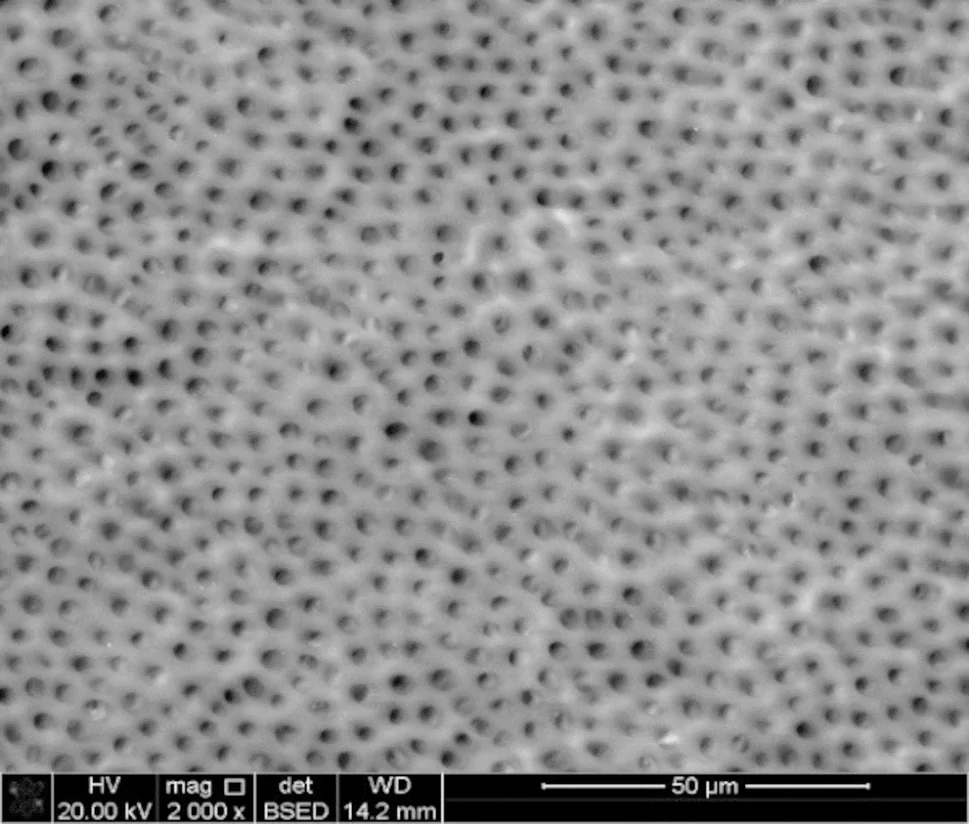
Representative ESEM photomicrograph (×2000) for dentin surface after hyaluronic acid treatment (M2) at T1 (after treatment), showing partially occluded and completely occluded dentinal tubules, Scale bar = 50 μm

**Fig. 4:**
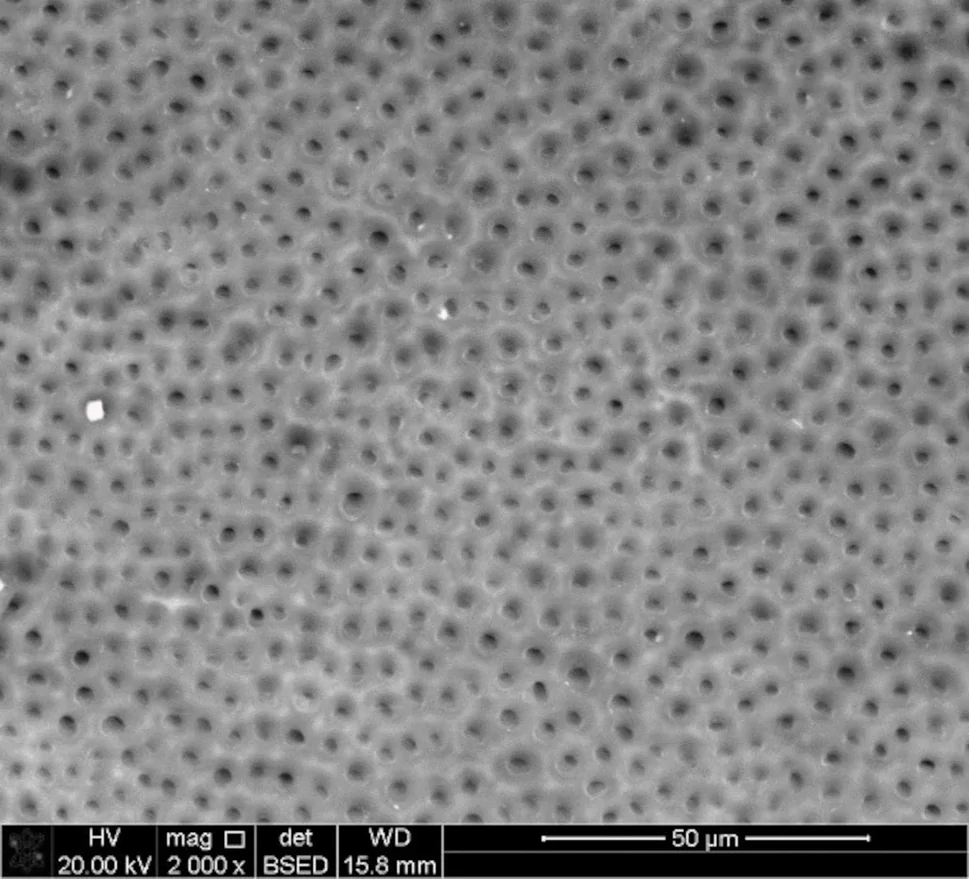
Representative ESEM photomicrograph (×2000) for dentin surface after diode laser application (M3) at (T1) (after treatment) with partially occluded dentinal tubules, Scale bar = 50 μm

**Fig. 5:**
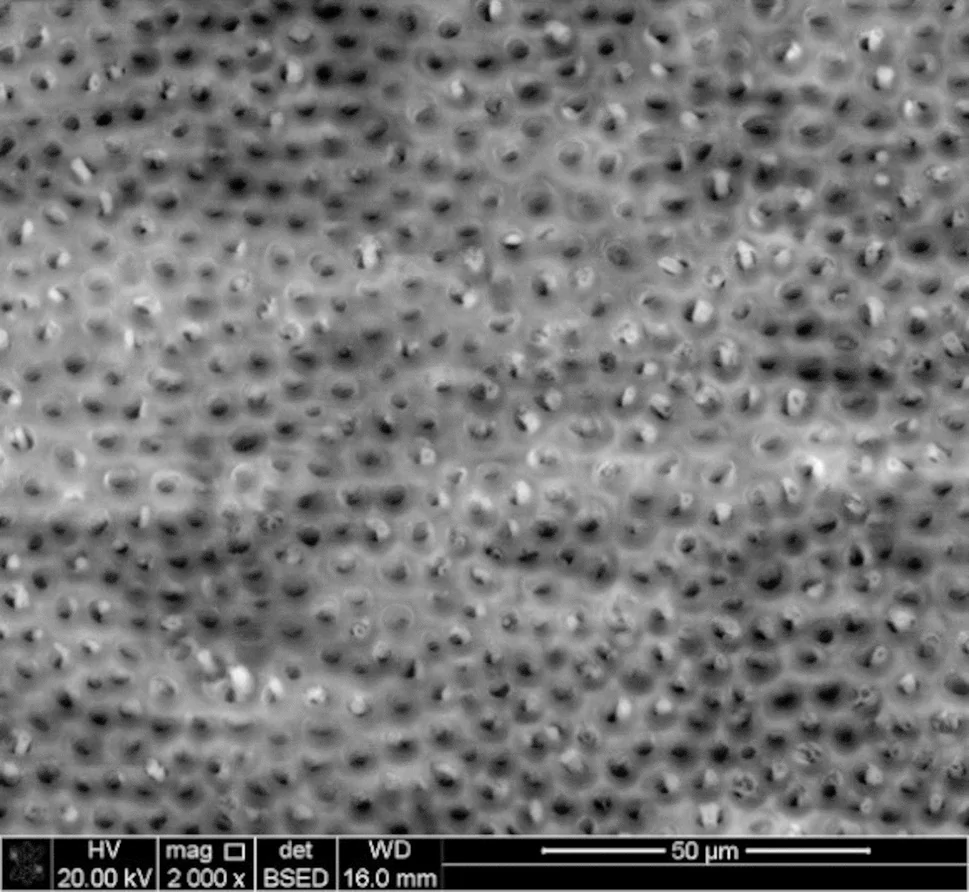
Representative ESEM photomicrograph (×2000) for dentin surface of hyaluronic acid + diode laser group (M4) at (T1) (after treatment) with partially occluded and fully occluded dentinal tubules, Scale bar = 50 μm

**Fig. 6:**
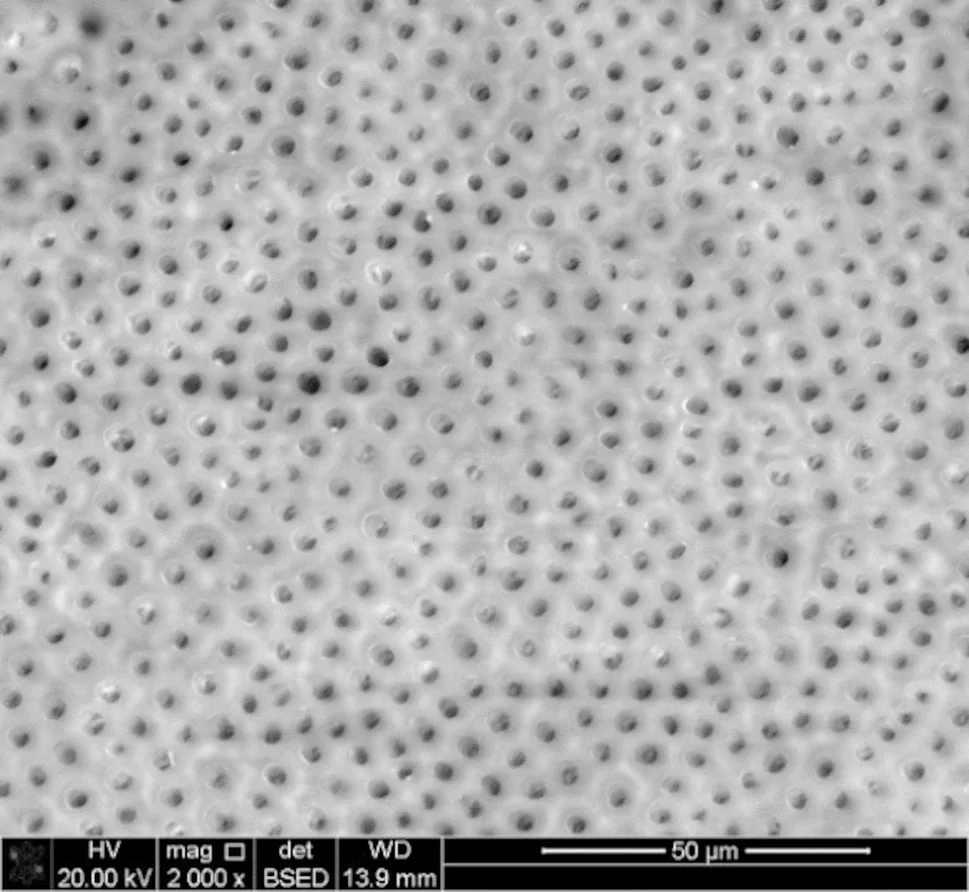
Representative ESEM photomicrograph (×2000) of the dentin surface in the hyaluronic acid + sodium fluoride group (M5) at T1, showing partially and completely occluded dentinal tubules. Scale bar = 50 μm

ESEM photomicrographs supported these findings. In the sodium fluoride group (M1), dentinal tubules appeared partially occluded with several patent tubules remaining (Fig. 2). In the hyaluronic acid group (M2), both partially and completely occluded tubules were observed (Fig. 3). The diode laser group (M3) showed partial occlusion consistent with laser-induced surface melting (Fig. 4). The hyaluronic acid + diode laser group (M4) demonstrated extensive tubule occlusion, with most tubules appearing partially or completely sealed (Fig. 5). Similarly, the hyaluronic acid + sodium fluoride group (M5) showed a high degree of tubule occlusion (Fig. 6).

After pH cycling (T2), all groups exhibited an increase in the surface area of open dentinal tubules, reflecting the effect of the acidic challenge. The highest mean value was again recorded in the sodium fluoride group (M1) (13.05 ± 1.55), followed by the diode laser group (M3) (10.06 ± 1.08), the hyaluronic acid group (M2) (8.68 ± 1.63), and the hyaluronic acid + sodium fluoride group (M5) (8.08 ± 1.71). The lowest mean value remained in the hyaluronic acid + diode laser group (M4) (6.45 ± 1.15), indicating superior resistance to demineralization. Statistical analysis demonstrated significant differences between M1, M3, and M4, while no significant difference was observed between M2 and M5 (*p* < 0.001).

**Fig. 7:**
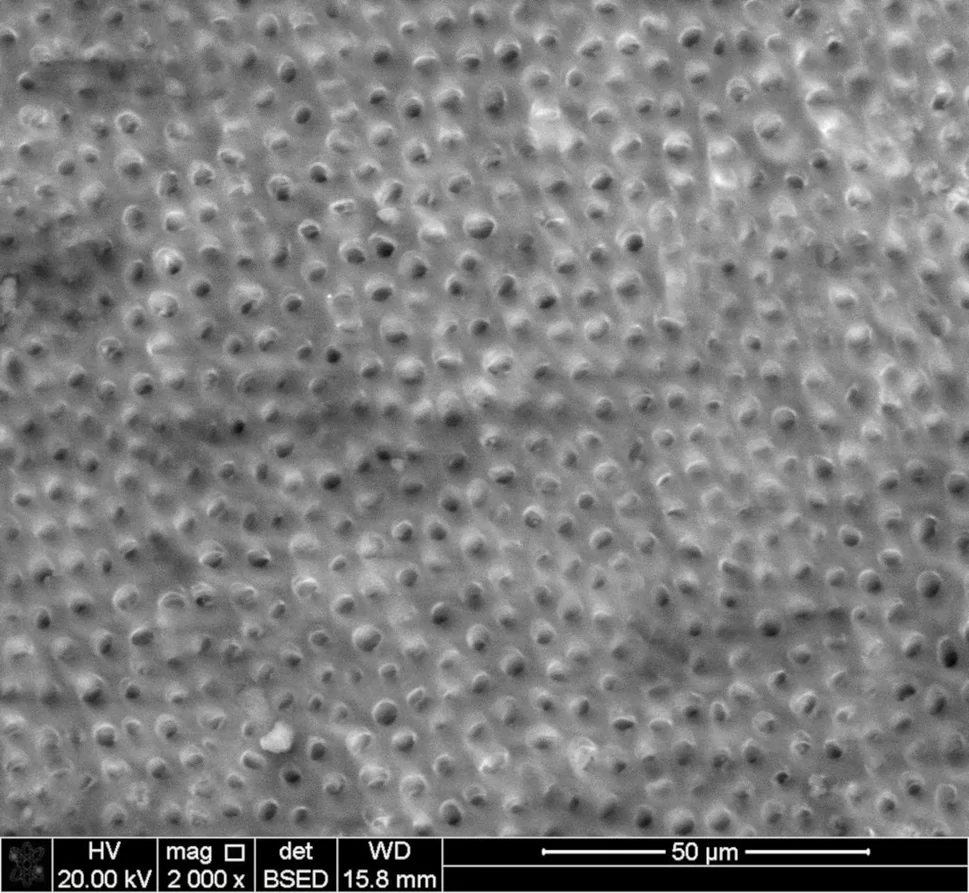
Representative ESEM photomicrograph (×2000) for dentin surface after pH cycling (T2), Scale bar = 50 μm

ESEM photomicrographs at T2 revealed partially occluded dentinal tubules with wider lumens compared to T1 across all groups, indicating partial loss of the occluding effect following pH cycling (Fig. 7).

## Discussion

Dentin hypersensitivity (DH) is a common oral condition which has a prevalence in the population [22]. The hydrodynamic theory is the most credible widely accepted scientific explanation for DH. Occluding the open dentinal tubules through formation of precipitates is an effective approach in the treatment of such condition [24]. One of the most used materials for occlusion of DT is sodium fluoride, which works through depositing crystals of calcium fluoride (CaF2) on the dentinal tubule opening with a short-term effect due to dissolution in saliva [25]. However, there are many other concerns such as fluorosis, gastric disturbance and toxicity so, the effect of other materials has been studied [26]. Hyaluronic acid (HA) is a natural structural element in many living tissues, which plays an important role in their development and regeneration. Also, it has a crucial role in intrafibrillar remineralization, so it may offer potential benefits in dentin hypersensitivity management; however, clinical evidence remains limited. Furthermore, Diode lasers treat DH by reducing nerve transmission and dissolving the dentin structure. Laser-induced surface modifications may improve dentinal tubule occlusion and could enhance the retention of desensitizing agents when used in combination. However, evidence supporting a definitive synergistic effect remains limited. So, the current study was directed to evaluate the effect of diode laser, hyaluronic acid, the combination of them, and the combination of sodium fluoride and hyaluronic acid on occlusion of dentinal tubules.

The use of 0.2% thymol-stored distilled water and artificial saliva may have influenced the degree of dentinal tubule stability and mineral deposition observed in the present study. Although these media are commonly used to preserve extracted teeth and simulate the oral environment, they do not fully replicate the dynamic biological conditions of the oral cavity, including salivary flow, enzymatic activity, and biofilm presence. This may have contributed to a more controlled environment that potentially enhances the observed occluding effects of the tested materials compared to clinical conditions. It should also be considered that the methodological choices used in this in vitro study may have influenced the observed outcomes. The standardized EDTA etching ensured complete smear layer removal and uniform exposure of dentinal tubules, which may have enhanced the apparent effectiveness of all tested agents compared to clinical conditions. Moreover, the absence of pulpal pressure and the use of static storage media could have reduced natural fluid flow within dentinal tubules, potentially overestimating the degree of tubule occlusion.

The present study aimed to evaluate the effect of different treatment modalities on dentinal tubule occlusion, with particular emphasis on the potential benefit of combination approaches. Rather than merely comparing numerical outcomes, the findings can be interpreted in light of the underlying mechanisms of each modality. The absence of significant differences among groups at baseline confirms the effectiveness of the standardization protocol, ensuring comparable initial conditions. This is consistent with previous in vitro studies that emphasized the importance of smear layer removal and uniform tubule exposure to allow valid comparison between treatment modalities [27].

Following treatment (T1), the observed differences among groups can be explained by the distinct mechanisms of action of the tested agents. Sodium fluoride demonstrated the least occluding effect, which may be attributed to the formation of a superficial calcium fluoride layer that is loosely bound and susceptible to dissolution. This is consistent with previous reports describing the limited durability of fluoride-based desensitizing agents under acidic conditions, in agreement with Kawle et al. [28]and Tanwar et al. [29].

In contrast, diode laser treatment showed improved tubule occlusion, likely due to thermal effects resulting in partial melting and recrystallization of the dentin surface. This mechanism has been reported in previous studies, where laser irradiation induces structural modification of peritubular dentin, leading to narrowing or sealing of dentinal tubules [30]. These results are in agreement with Baro et al. (2023) [31] and Sahoo et al. (2023) [32]., who reported that diode laser had a statistically significantly greater effect on dentinal tubule occlusion compared with NaF. Similarly, George et al. (2019) [33] evaluated the effects of diode laser and other desensitizing agents on dentinal tubule occlusion and found a highly significant difference between groups, with the diode laser group showing superior results.

Hyaluronic acid demonstrated a greater occluding effect compared to diode laser alone. This may be attributed to its role in dentin matrix development and induction of mineralization and odontogenic differentiation. Also, it was reported that it plays an important role in intrafibrillar remineralization [9]. Interestingly, the combination groups exhibited superior performance compared to individual treatments. This improvement can be interpreted as a result of complementary mechanisms rather than a confirmed synergistic interaction. In the hyaluronic acid and diode laser group, laser-induced surface modification may have created a more retentive substrate, facilitating deeper penetration and stabilization of hyaluronic acid within dentinal tubules. This explanation is consistent with studies reporting enhanced material retention following laser irradiation. The present results are in agreement with Gouda et al. (2021) [34], who demonstrated that the combination of a 980 nm diode laser and nano-hydroxyapatite (nHAP) produced a significantly higher percentage of dentinal tubule occlusion compared with either treatment alone. Similarly, Shamel and El Banna (2022) [30], reported that combining diode laser with nHAP toothpaste was more effective in occluding dentinal tubules than the individual modalities. In addition, Tanwar et al. (2022) [29] found that the use of a 980 nm diode laser in combination with sodium fluoride resulted in a significant reduction in tubular diameter compared with single-agent treatments. Consistent findings were also reported by Doppalapudi et al. (2023) [35], who observed superior dentinal tubule occlusion when diode laser was combined with desensitizing toothpaste.

On the other hand, the present findings are in disagreement with Sameera et al. (2019) [36], who evaluated the efficacy of strontium chloride, Biodentine alone, and Biodentine in combination with a diode laser on dentinal tubule occlusion and reported that Biodentine alone produced better results than the combination group. This discrepancy may be attributed to differences in the laser application technique, as the authors used a contact mode, which may have led to partial removal of the Biodentine layer from the dentin surface during laser irradiation. Another possible explanation is the difference in the occluding agents used between studies.

After pH cycling (T2), all groups exhibited an increase in the surface area of open dentinal tubules, reflecting the impact of acidic challenge. Nevertheless, the combination of hyaluronic acid and diode laser maintained the lowest values, suggesting improved resistance to demineralization. This may be attributed to the formation of a more stable and integrated occluding layer, combining structural modification of dentin with bioactive remineralization.

The present results are in agreement with Gouda et al. (2021) [34], who reported that the combination of a 940 nm diode laser and nano-hydroxyapatite (nHAP) provided the greatest resistance to acidic challenge compared with either treatment alone. Similarly, Shamel and El Banna (2022) [30], found that the combined application of diode laser and nHAP toothpaste resulted in greater resistance to acid challenge than the individual treatments.

In contrast, the greater increase observed in sodium fluoride and hyaluronic acid groups may indicate that their effects are primarily surface-based and more susceptible to dissolution. Based on all the previously mentioned results, the null hypothesis of the current study was partially rejected.

Despite the standardized methodology, certain limitations should be acknowledged. Examiner calibration and inter-/intra-examiner reliability assessment were not performed; however, a single examiner conducted all measurements using a standardized ImageJ protocol to reduce variability. As an in vitro study, the experimental conditions do not fully replicate the oral environment, particularly salivary dynamics, pulpal pressure, and biofilm interactions, which may influence clinical behavior. In addition, the standardized single-application protocol and fixed exposure times may not fully reflect clinical variations in material application. The present study also focused solely on surface examination of dentinal tubules and did not assess the depth or actual penetration of the desensitizing agents, which is an important factor for long-term relief of dentin hypersensitivity. Furthermore, dentin permeability was not evaluated. Therefore, the results should be interpreted with caution, and further in vivo studies are recommended to confirm these findings.

## Conclusions

Within the limitations of this in vitro study, all tested modalities were effective in reducing the surface area of open dentinal tubules to varying degrees. The combination of hyaluronic acid and diode laser (M4) demonstrated the most pronounced occluding effect, indicating superior performance compared with all other groups. Notably, hyaluronic acid alone exhibited greater efficacy than diode laser treatment. The enhanced effect observed with the combination approach may be attributed to complementary mechanisms of action and suggests a potential synergistic interaction, although this was not directly evaluated. However, given the surface-based and laboratory nature of the assessment, further in vivo studies are required to confirm the clinical relevance and long-term stability of these findings.

### Recommendations

1. Further in vivo studies are needed to assess the efficacy of diode laser and hyaluronic acid in reducing dentin hypersensitivity.
2. Further studies are needed to assess the depth of penetration of the agents into the dentinal tubules.

## Supplementary Information

Supplementary Material 1: Supplementary Figure S. Representative ImageJ analysis workflow for dentinal tubule assessment demonstrating image thresholding and segmentation used to quantify open dentinal tubule area.

## Data Availability

The datasets used and/or analyzed during the current study are available from the corresponding author upon reasonable request.

## Abbreviations

DH: Dentin hypersensitivity
DT: Dentinal Tubules
ANOVA: Analysis of variance
ESEM: Environmental Scanning electron microscopy
